# Can African elephants use leaf colour as a visual cue when making foraging decisions?

**DOI:** 10.1007/s10071-025-01972-z

**Published:** 2025-06-13

**Authors:** Claire L. Peinke, Adrian M. Shrader

**Affiliations:** Department of Zoology & Entomology, University of Pretoria, Private Bag X20, Pretoria, 0028 South Africa

**Keywords:** Choice experiments, Distance, Foraging decisions, *Loxodonta africana*, Patch choice, Vision

## Abstract

Finding food is one of the most important aspects of an animal’s life. Yet, locating it can be challenging as the availability and quality of food varies both spatially and temporarily. To overcome these challenges, mammalian herbivores may use spatial memory or senses such as smell and vision. Recent studies have found that African elephants (*Loxodonta africana*) use olfactory cues to locate food patches and to select plants to eat within these patches. However, the extent to which they may also use visual cues, such as those associated with leaf colour, a proxy for food quality, when making foraging decisions is unknown. To address this knowledge gap, we tested whether African elephants were able to discriminate differences in leaf colour (i.e., light green: high-quality new growth, dark green: lower quality old growth, brown: poor-quality senesced vegetation) over a range of distances (i.e., 3, 5, 10, 20, 40, 80 m). Testing four semi-tame elephants using visual-based choice experiments between different coloured canvases, we found that the elephants were able to discriminate colour differences at close range (i.e., *≤*10 m), but not beyond. This suggests that when feeding within a patch, African elephants may utilise the visual cues associated with leaf colour to help locate high-quality food (e.g., individual trees, branches, and leaves) and thus increase their foraging efficiency. However, as they were unable to discriminate between the different colours over distances > 10 m, it is unlikely that they use colour when making long-range foraging decisions such as selecting feeding patches.

**Supplementary Information:**

The online version contains supplementary material available at 10.1007/s10071-025-01972-z.

## Introduction

A key challenge of any animal’s life is locating food. This is especially true for mammalian herbivores where the quality and availability of vegetation varies both spatially and temporally with the changing seasons (Grunow et al. 1980; Shrader et al.^ 2006^). Nevertheless, these herbivores can use a range of cues to locate food such as odour and visual signals. Colour vision can be beneficial while foraging as it can help animals select high-quality food and food patches. For example, Belding’s ground squirrels (*Urocitellus beldingi*) utilise flower colour to locate preferred plants (Eshelman and Jenkins 1989; Duncan and Jenkins 1998). By using colour as a cue, these squirrels can detect food from a distance and thus forage more efficiently. In addition, cattle can visually discriminate between green (alive) and brown (dead) grass, which allows them to increase their nutritional intake (Hirata et al. 2019; Hirata and Kusatake 2020). However, cows are only able to make these colour discriminations over small spatial scales (i.e., 1–3 m).

Colour vision varies greatly across taxa (Jacobs 1983; Bowmaker 1998). It is highly developed in birds (Bowmaker 1980; Finger and Burkhardt 1994; Kelber 2019) and insects (Menzel 1979; Stavenga 2002; Warrant and Somanathan 2022), but very few mammals can detect a wide range of colours (Jacobs 1993, 2009; Jacobs and Rowe 2004). For example, the occurrence of dichromacy varies in the order of primates, with incidences of red-green colour blindness being shown within tamarins (*Saguinus* spp.), Geoffroy’s marmosets (*Callithrix geoffroyi*), long-tailed macaques (*Macaca fascicularis*) and chimpanzees (*Pan troglodytes*) (Caine et al. 2003; Dominy et al. 2003; Smith et al. 2003; Saito et al. 2005). In addition, elephant shrews (Order Macroscelidea) have been shown to have dichromatic colour vision as they are able to distinguish blue and green from different shades of grey at close distances (Thüs et al. 2020). Furthermore, both African (*Loxodonta africana*) and Asian (*Elephas maximus*) elephants have dichromatic colour vision, which prevents them from observing shades of red and green (Yokoyama et al. 2005). However, one of the evolutionary advantages of red-green colour-blindness is the ability to detect the contrast of colour-camouflaged foods, such as flowers and fruits that are similar in colour to background foliage (Judd 1943; Morgan et al. 1992; Regan et al. 2001).

As leaves develop, their colour tends to change. Specifically, new growth is generally light green, but as leaves mature, they become a darker shade of green (Lucas et al. 1998; Loarie et al. 2009). Then, as the leaves senesce, they turn brown due to the photodegradation of chlorophyll (Maunders and Brown 1983). Linked to these colour changes are changes in food quality for herbivores. The highest quality food is generally found in new growth as it tends to be low in fibre, and the ratio of secondary compounds to crude protein (i.e., nitrogen) is generally low (Thoma et al. 2002; Loarie et al. 2009; Shrader et al. 2012). However, as leaves develop, fibre content increases, as does the secondary compound to crude protein ratio (Choong et al. 1992; Lucas et al. 1995), which lowers the nutritional content. Finally, when plants senesce and turn brown, they reabsorb nutrients from the dying leaves, which greatly reduces leaf quality for herbivores (Bloom et al. 1985; Lajtha 1987; Aerts 1996; Killingbeck 1996).

Due to the link between colour and leaf quality (Macandza et al. 2004; McNaughton 2013), it would make evolutionary sense for herbivores to use colour cues when deciding on what to eat. Moreover, depending on from how far away they can distinguish between these colours, they may use canopy colour as a visual cue to determine between and within patch foraging decisions. If this is the case, then the use of colour cues would help reduce a herbivore’s search time while foraging, likely increasing daily nutritional intake, and ultimately foraging efficiency. However, as some mammals are dichromatic, their perception of leaf colour would differ to our own (Yokoyama et al. 2005). Nevertheless, they may still associate visual cues linked to these colours (e.g., contrast, brightness) to food quality (Judd 1943; Morgan et al. 1992; Regan et al. 2001).

To explore the extent to which African elephants may use visual cues associated with leaf colour when making both between and within patch foraging decisions, we conducted a series of visual-based choice experiments with four semi-tame adult African elephants. Using coloured canvases, we tested the distance at which these elephants could discriminate between visual cues associated with different leaf colours (i.e., light green, dark green, brown). Due to the foraging benefits (e.g., increased nutritional gain, greater foraging efficiency) that African elephants would gain from using the visual cue of leaf colour as a visual cue, we predicted that they would be able to discriminate between these colours. In addition, we predicted that as distance increased the elephants’ ability to discriminate between the colours would decrease. Alternatively, since making foraging decisions over larger spatial scales would be beneficial with regards to reducing search time and increasing daily intake rate, it was possible that the elephants would be able to discriminate the visual cues associated with colour differences over the full range of distances. Finally, it may be that the colour-limited vision of elephants prevents them from using colour as a visual cue while foraging across the different distances.

## Methods

### Sampling

Our study was conducted at Adventures with Elephants, Bela Bela, South Africa from mid-August to mid-October 2022. This facility holds Performing Animal Protection Act (PAPA) and Threatened or Protected Species (TOPS) permits. Study animals comprised four semi-tame adult (ages 22 to 30 years) African elephants (3 females, 1 male). The heights of these individuals (i.e., 2.45–3.50 m at the shoulder) are not drastically different and are therefore unlikely to result in differences in visual range and thus inter-individual variation. This apparently small sample size if offset by the fact that semi-tame African elephants are very rare. As such, these individuals represent a relatively large sample size under the circumstances. All procedures were conducted by the elephants’ experienced handlers to ensure that the animals’ wellbeing was maintained. The training phase was conducted over ~ 6 weeks and the experimental phase for ~ 2 weeks (see details below). Before the training and final experiments were conducted in the morning, the elephants were given water, but no food so as to increase their interest in taking part in the experiments. After which, they foraged across the landscape feeding on natural vegetation (grass and woody plants), as well as being provisioned with wild game pellets (ALZU Feeds, South Africa) during interactions with guests.

## Experimental procedure

**Fig. 1:**
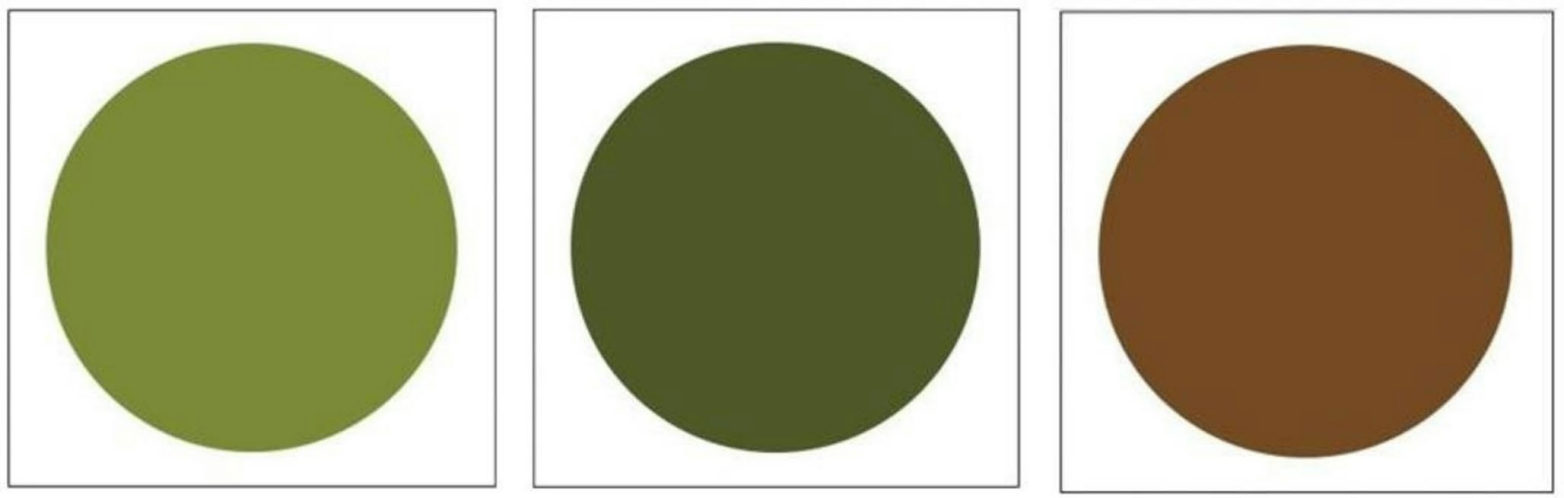
The three 1.2 m x 1.2 m leaf colour canvases each with a circle of 1 m in diameter. Colours represent new growth (light green), old growth (dark green), and senesced (brown) leaves of the fruit bush willow (*Combretum zeyheri*)

To determine the extent to which elephants may use the visual cue of leaf colour when making foraging decisions, we conducted a series of visual-based choice experiments. For the experiments, we had the elephants make choices between three 1 m diameter circles of different colour printed on separate 1.2 m x 1.2 m canvases (Fig. 1). The colours represented the different stages of leaf development (i.e., light green: new growth, high crude protein to fibre ratio; dark green: old growth, lower crude protein to fibre ratio compared to new growth; brown: senesced vegetation, low quality, high fibre; Loarie et al. 2009). We extracted these colours from images of fruit bush willow (*Combretum zeyheri*) leaves using Canva– Colour Palette Generator (https://www.canva.com/colors/color-palette-generator/*).* We found the images by searching “fruit bush willow leaves” on Google. We used the colour of fruit bush willow leaves as it is both a preferred and principal species in the diet of the elephants used in this study (see Schmitt et al. 2016; Schmitt et al. 2018).

**Fig. 2:**
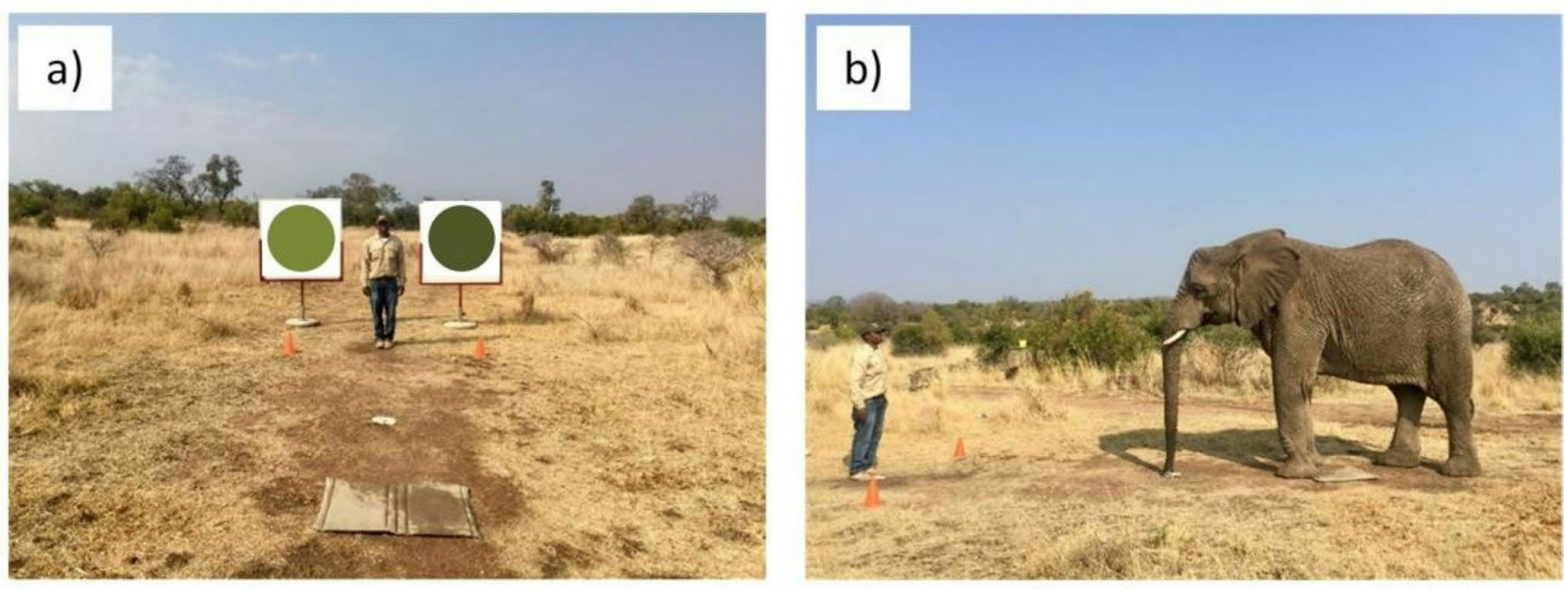
The experimental set up **(a)** from the elephants’ perspective showing the mat used as the starting position, the two orange cones, the handler, and the 1.2 m x 1.2 m canvases (light green on the left, dark green on the right) sitting in the metal frames 5 m from the starting position, and **(b)** a side view with an elephant and the handler that gave the commands standing at the starting position

To conduct the experiments, one handler stood in front of the elephant being tested and instructed it to stand with its front feet on a mat placed on the ground and the tip of its trunk touching the ground (i.e., the starting position; Fig. 2). Three metres in front of the mat, were two small (~ 30 cm tall) orange cones (one on the left and one on the right) separated by three metres. Each canvas was inserted into a free-standing metal frame that was located at a specific distance behind the cones (see details below). These frames held the canvases 70 cm above the ground (Fig. 2a). This allowed the elephants to view the canvases without the canvases having to be held by the handlers. By using frames for the canvases, we eliminated the possibility of the elephants making choices based on the handler holding a canvas and not the colour of the circle on the canvas. To give the verbal commands, the handler in front of the elephants stood between and a little behind (~ 50 cm) the cones during the experiments (Fig. 2a). To prevent the handler from unconsciously signalling to the elephant, we did not let the handler know which side the higher-quality colour circle was located.

Once the elephant was at the starting position, the handler verbally instructed the elephant to choose one of the printed canvases from the dyad comparison (i.e., the comparison of two printed canvases) in front of them. To do this, the elephant walked forward and touched the cone in front of the canvas they wanted (i.e., right cone indicated right canvas, left cone indicated left canvas; Fig. 2a). During the experiments, if the elephant chose the canvas with the higher-quality colour (i.e., light green > dark green, light green > brown, dark green > brown) in the dyad comparison, they were given 100–125 g (i.e., four handfuls) of wild game pellets (ALZU feeds, South Africa) as a food reward (i.e., positive reinforcement). This amount of food provided is comparable to a medium trunkful of food that elephants take while feeding (Schmitt et al. 2016). If, however, the elephant chose the lower quality circle in the dyad comparison, they did not receive a food reward. After making their choice and receiving the reward (or not), the elephant was then verbally instructed to back up to the starting position.

To test the elephants’ ability to distinguish differences in colour with increasing distance, we used the same experimental procedure, but the elephants were tested with the canvases set up at 3, 5, 10, 20, 40 and 80 m away from the starting position. For every 1 m the canvases were moved away from the elephants, the distance between the canvases was increased by 0.14 m. This resulted in the distances between the canvases being 3.0 m at 3 m, 3.3 m at 5 m, 4.0 m at 10 m, 5.4 m at 20 m, 8.3 m at 40 m, and 14.0 m at 80 m. We did this to make it easier for the elephants to distinguish between the canvases with increasing distance. Yet, despite the canvases being 14 m apart at 80 m from the starting position, both canvases were still within the elephants’ field of vision (313°) (Suedmeyer 2006) such that they did not have to move their head from side-to-side to be able to see both canvases.

All choice experiments were conducted at 08:00. For logistical reasons, during a session, all four of the elephants were tested at the same distance using one dyad comparison, which resulted in five tests per elephant per session. This totalled 90 tests per elephant conducted over 18 days. This is calculated as each elephant was tested five times per combination (new growth vs. old growth, new growth vs. senesced, senesced vs. old growth) at each of the six distances (3, 5, 10, 20, 40, 80 m). However, the order in which the elephants were tested, the order of the colour comparisons (i.e., light green vs. dark green, light green vs. brown, dark green vs. brown), and the order of the distances at which the elephants were tested (i.e., 5, 10, 40, 3, 20, 80 m) were determined randomly. Moreover, when we tested an individual, we determined the initial position of the canvases, either left or right, randomly with a coin toss. As such, we are confident that the extent to which we randomised the majority of the factors was more than sufficient to prevent order effects.

Once an elephant had made its choice, the handler had the elephant back up and turn so that it faced away from the canvases. While the elephant was facing the opposite direction, a coin was flipped to determine whether the position of the canvases should be swapped (e.g., heads = swap, tails = no swap). A “fake swap” was conducted if the canvases were meant to stay in place. This is where two handlers each picked up one of the canvases, walked towards each other until they met in the middle between the frames and then walked back to their original position. This simulated the sound and time it took to swap the canvases. This prevented the elephants from potentially using time or sound as an indicator of the canvases being swapped, and thus knowing where the high-quality colour was located without having to look.

Once the canvases were back in place, the handler verbally instructed the elephant to turn and face the canvases, stand at the starting position, and then make their choice. To ensure that the elephant being tested did not cue off any mucous deposited from the previous elephant (i.e., olfactory cues), the cones were wiped with a damp cloth after each test. In addition, the cones were cleaned with soap and warm water, and allowed to dry in the sun after each session. This ensured that any lingering odours deposited by any of the elephants from the previous session were removed.

When we tested the elephants at a distance > 5 m (i.e., 10, 20, 40 and 80 m), we first ran three tests at 5 m to remind the elephant of the experimental design. The canvases were then moved to the correct distance while the elephant was watching, but we had the elephant look away while we determined which side the canvases should be placed. We did this as we were unsure how good the elephants’ vision was, and thus wanted to ensure that they knew where the canvases were placed for these more distant trials. We then ran the tests at these farther distances as described above.

## Training

Prior to conducting the experiments, we trained the elephants to understand the experimental procedure and what each of the canvases represented. This was done by first getting them to understand that selecting the higher-quality colour circle (i.e., light green > dark green, light green > brown, dark green > brown) would result in them getting a food reward (i.e., positive reinforcement), while selecting the lower quality circle would result in them not receiving anything. Due to the small sample size, we did not counterbalance the design by training half of the elephants to select for the lower-quality vegetation. These initial training experiments were conducted with the canvases 5 m away from the starting position, and having the elephants walk up and touch the canvas with their trunk to make their choice.

Once all the elephants were consistently selecting the highest quality colour canvas, we then introduced the cones and trained the elephants to make the link between touching a cone and selecting the canvas associated with that cone. For the cone training experiments, the canvases were placed 5 m away from the starting position and the two cones were placed 3 m in front of the canvases. The elephants were then verbally instructed to walk up and touch one of the cones with their trunk. Similar to when we trained the elephants to understand the differences between the colours, if the cone they selected corresponded with the higher-quality colour canvas, we gave them a food reward, but we gave them nothing if they selected the cone corresponding to the lower quality colour canvas. We ran the training trials for thirty days, and once the elephants were consistently selecting the cone associated with the higher-quality canvas, we collected and analysed the data to determine whether the elephants understood the experimental design (i.e., significantly selected the higher-quality colour canvas) prior to running the experiments (see Figure OR1, Online Resource 1). Having determined that the elephants could significantly make the correct choices (Generalized Estimating Equation: χ^2^ = 12720912.62, df = 2, *p* < 0.0001), we stopped the training period and started collecting data across all the distances as described above.

### Data analysis

To determine whether the elephants understood the experimental procedure and could tell the difference between the canvases during the training period, we used Generalized Estimating Equations (GEEs) with an exchangeable correlation matrix and binomial error distribution with a logit link function. We did this as the data comprised a set of binary choices, with each elephant being tested multiple times (i.e., repeated measures; Ballinger 2004) (see Schmitt et al. 2018 and Wood et al. 2022 for a similar procedure). However, despite collecting repeated measures, our small sample size did not allow us to generate meaningful results on the variation of choices made by the individual elephants, and thus we did not employ mixed-effects models (Ma et al. 2012; Naseri et al. 2016). Rather, we used GEEs which focus on population-level responses, compensate for nonindependence in the data, and can handle small sample sizes (Ballinger 2004; Ma et al. 2012; Naseri et al. 2016). In our study, the GEEs modelled the overall average proportion of the elephants making a specific choice and compared that proportion to the expected random selection of 50%. For the training analysis, we defined the subject variable as the individual elephants and the within-subject variable as the week of training (weeks 1–4). These training trials ran for thirty days (i.e., 4 weeks; *N* = 1144 trials).

We also used GEEs for the main experiments to determine the elephants’ overall ability to discriminate between the different colours across the different distances. As in the previous analysis, the GEEs incorporated an exchangeable correlation matrix and binomial error distribution with a logit link function. We defined the subject variable as the individual elephants, and the within-subject variables as the combination of colour choices presented to the elephants, the distances at which these choices were presented, and the interaction of colour and distance.

GEEs model the proportion of the choices made by the elephants between the different colours, with the p-value indicating whether the variable influenced the choices made. For example, whether the elephants consistently chose the highest quality colour across the different distances. To determine the specific choices at the different distances, we back transformed the data from the logit-scale for graphical representation. We then used the estimated marginal means and the asymmetrical 95% confidence intervals (CIs) to determine whether the elephants’ choices differed to the expected 50% random selection (i.e., CIs not overlapping the 50% line). If the CI were above the 50% line, it indicated that the elephants consistently selected the colour representing the higher-quality leaves in the comparison. However, if the CI overlapped the 50% it indicated random selection (i.e., guessing), while CIs below the 50% line indicated the elephants consistently selected the colour representing the lower-quality leaves. All analyses were conducted using IBM SPSS 29.0.0.0. (241) (IBM Corp. 2020).

## Results

**Fig. 3:**
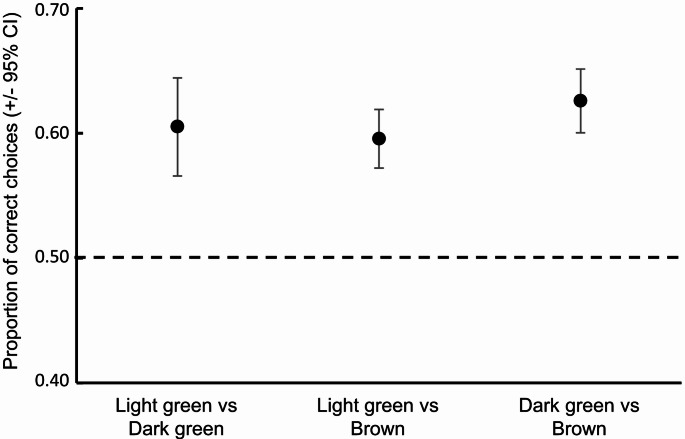
Proportion of choices made by the elephants for the higher-quality leaf colour in the different dyad comparisons. Marginal means (+ 95% CI) for the proportion of selection for each option are plotted. Overlap of the error bars with the expected of 0.5 (dashed line) indicates random selection (i.e., no preference). Error bars above the 0.5 expected indicates selection of the higher-quality leaf colour, while error bars below the line indicates selection of the lower-quality leaf colour

**Fig. 4:**
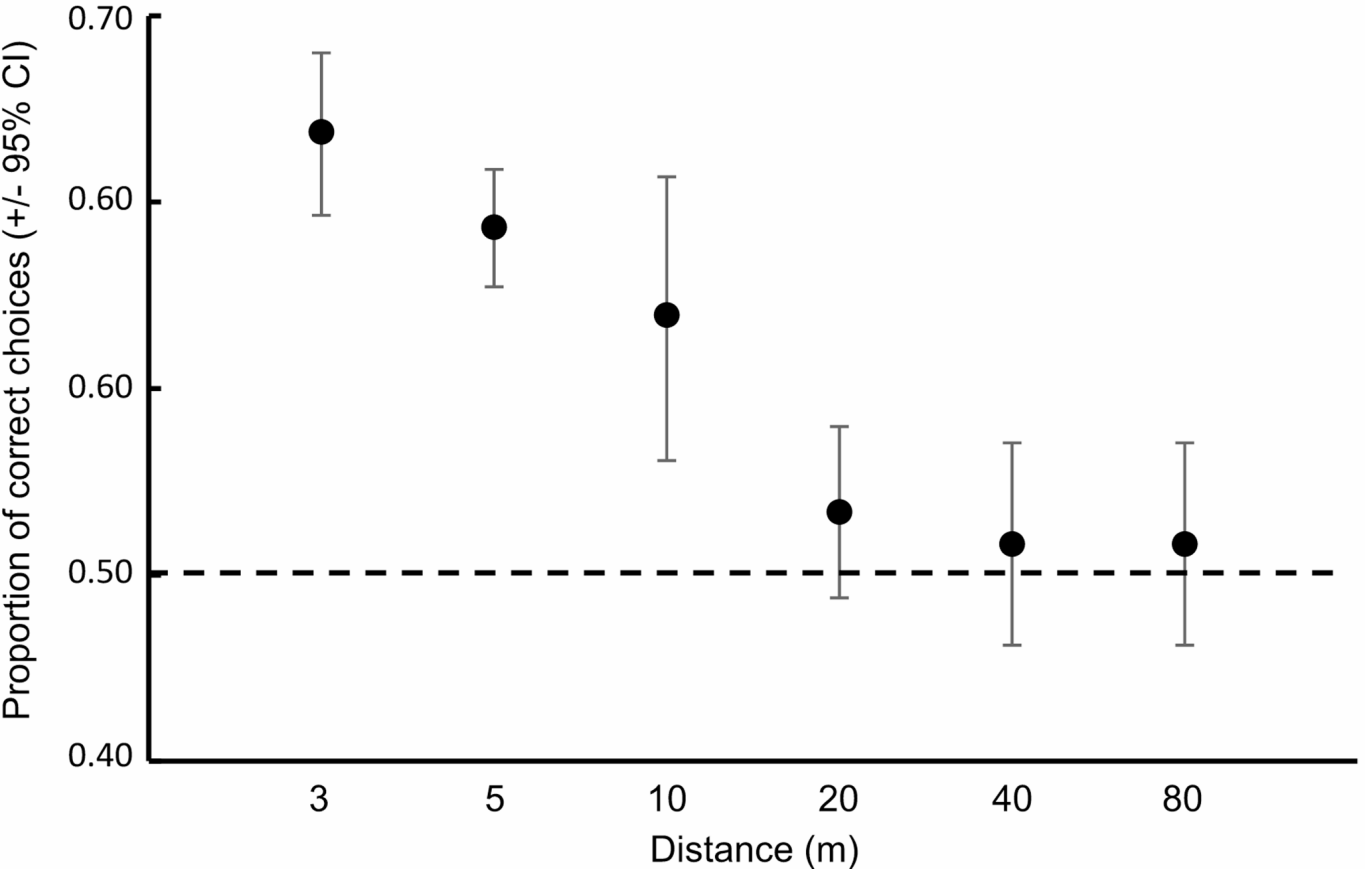
Proportion of correct choices (i.e., for the high-quality colour) made by the elephants at the different distances. Marginal means (+ 95% CI) of the proportion of selection at each distance are plotted. Overlap of the error bars with the expected 0.5 (dashed line) indicate random selection (i.e., no preference). Error bars above the 0.5 expected line indicate selection of the ‘correct’ high-quality leaf colour, while error bars below the line indicate significant selection for ‘incorrect’ lower-quality leaf colour

**Fig. 5:**
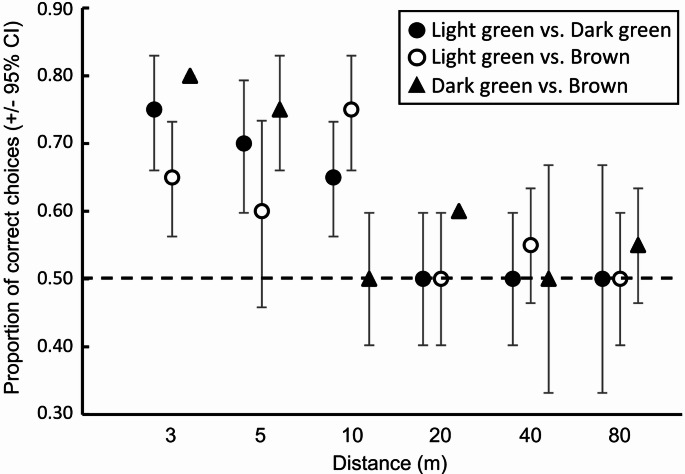
Proportion of correct choices made by the elephants for the highest-quality leaf colour in the three comparisons (i.e., light green vs. dark green, light green vs. brown, and dark green vs. brown) at the different distances. Marginal means (+ 95% CI) of the proportion of selection for each option are plotted. Overlap of the error bars with the expected 0.5 (dashed line) indicate random selection (i.e., no preference). Error bars above the 0.5 expected choice for the high-quality leaf colour, while error bars below the line indicate preference for the lower-quality leaf colour

The elephant’s ability to select the highest-quality leaf colour differed between the different leaf colour combinations (GEE: χ^2^ = 9.251, df = 2, *p* = 0.010). However, the elephants consistently selected the highest-quality colour across all three combinations (Fig. 3). Yet, their ability to make correct decisions varied with distance (GEE: χ^2^ = 89.929, df = 3, *p* < 0.001), with the elephants only being able to consistently select the highest-quality colour at distances *≤* 10 m (Fig. 4). At the greater distances (i.e., > 10 m) the elephants’ selection was random indicating that they were unable to distinguish between the different colours at these greater distances (Fig. 4). This same pattern was relatively consistent across all three colour combinations at the different distances (GEE: χ^2^ = 129.779, df = 3, *p* < 0.001; Fig. 5), with the only exceptions being at five metres where the elephants randomly selected between light green and brown, and at 20 m where they were able to consistently select dark green over brown. Nevertheless, the elephants never displayed a consistent preference for any of the lower quality colours across the different distances (Fig. 5). Throughout the experiment, each individual elephant did make “mistakes” in that they periodically selected the lower-quality colour. As such, the overall pattern observed was not driven by one individual consistently making “mistakes”, while the others consistently made correct choices (i.e., inter-individual variation).

## Discussion

Colour vision is important in many aspects of animals’ lives including mate choice (Bennett et al. 1996; Andersson and Amundsen 1997; Changizi et al. 2006), predator detection (de Moraes et al. 2021, 2023), and foraging (Sumner and Mollon 2000; Melin et al. 2017a, b). As colour can indicate the ripeness of fruit (e.g., Medlicott et al. 1986; Cox et al. 2004; Mikulic-Petkovsek et al. 2015) and the nutritional quality of vegetation (Macandza et al. 2004; McNaughton 2013), it would make evolutionary sense that mammals would use colour cues to help make foraging decisions. However, the extent to which African elephants may use colour as a visual cue, and the distance at which they can discern colour differences, is unknown. Despite African elephants being dichromatic (i.e., red-green colour blind; Yokoyama et al. 2005), we found that when we presented them with the different leaf colours (i.e., light green, dark green, brown), the elephants were able to discriminate between them, and select the highest-quality colour at close distances (i.e., 3, 5 and 10 m). However, at distances > 10 m (i.e., 20, 40 and 80 m), the elephants’ choices became random, suggesting poor distance vision (Suedmeyer 2006).

The fact that the elephants in our study were unable to discriminate between the colours at distances > 10 m suggests that they have better close vision, compared to distance vision (i.e., they are near sighted). This is similar to previous studies which highlight that both African and Asian elephants have better close distance vision. For example, one Asian elephant was able to visually discriminate amongst various patterns at close distances (Rensch 1956, 1957). Furthermore, African elephants have been shown to be able to discriminate small objects of about 2.75 cm at a distance of 196 cm (Shyan-Norwalt et al. 2010) as well as discriminate differences in objects at a distance of 1.5 m (Savage et al. 1994). Moreover, previous research found that as distance increases, mammals’ visual ability decreases (Johnsen 2012; Caves et al. 2018). As such, it is unlikely that African elephants use colour cues when deciding between distant feeding patches (> 10 m). Rather, they likely rely on other cues such as odour to reduce search time while feeding across the landscape (Schmitt et al. 2018). However, since our canvases represent relatively small trees and bushes, it is still possible that elephants may be able to use colour when deciding between large patches of trees with canopies greater than 1 m in diameter. This, however, requires further study.

Despite the overall pattern of the elephants being able to discriminate between the colours at *≤* 10 m but not beyond, there were a few exceptions. Specifically, the elephants were able to differentiate between light green and brown at 3 m, but then unable to tell the difference between these two colours at 5 m. Yet, then at 10 m the elephants were once again able to discriminate between these colours. This is strange as it is unlikely that the elephants’ ability to discriminate between these colours would fluctuate in this manner over such a short distance. One possible reason for this are the wide confidence intervals for this comparison at 5 m, likely driven by a few more individuals making mistakes compounded by the small sample size.

The second exception is between dark green and brown. Looking at the pattern, it seems that the elephants’ ability to discriminate between these colours declines at 10 m, while they are still able to distinguish between the other colour combinations, both of which include light green. A decline in the ability to distinguish between these two darker colours makes sense as these darker colours may look similar at greater distances (e.g. have similar brightness). However, then at 20 m our results suggest that the elephants were once again able to discriminate between these two colours. We find this unlikely as the elephants are unable to discriminate between any other colour combination at this distance. Rather, it is likely the result of our small sample size. However, rerunning the experiments with a larger sample size would likely clear up these strange patterns.

By using visual cues at close distances (i.e., *≤* 5 m), elephants are likely able to maximise their nutritional gain and feeding efficiency when feeding within a patch. Calculating the visual acuity of African elephants, Pettigrew et al. (2010) suggested that African elephants would be able to discriminate between potential food items as small as 1 mm within the distance that they can reach with their trunk. Thus, as African elephants are able to see over the 5 m distance that they can reach with their trunks (Stone and Halasz 1989; Pettigrew at al. 2010; Shyan-Norwalt et al. 2010), they are likely able to visually select specific branches containing higher quality leaves, and specific green leaves on these branches (Pettigrew at al. 2010). This is important since despite their large body size, and thus an ability to tolerate low-quality food, African elephants feed selectively (Owen-Smith and Chafota 2012; Shrader et al. 2012). Specifically, they prefer leaves over other plant parts, such as branches and bark (Owen-Smith and Chafota 2012; Seloana et al. 2017) and higher quality leaves compared to low-quality ones (Jachmann 1985; Pretorius et al. 2012; Owen-Smith and Chafota 2012). Therefore, the ability to discriminate between leaf colour likely helps elephants to select the highest quality plants and plant parts within a patch.

Despite our study focusing on colour, one aspect that we did not consider in our experimental design were potential differences in the brightness of the different colours printed on the canvases (Fairchild, 2005; Corney et al. 2009). In general, visual acuity and brightness are positively correlated with brighter objects being more easily seen (Shlaer 1937; Hecht 1949). As such, it is possible that instead of using colour as a cue in our experiments, the elephants may have rather used differences in the brightness of the colours when making their choices. For example, the light green canvas was likely brighter than the dark green and brown canvases. However, the extent to which the brightness of the dark green and brown may have differed is uncertain. Similarly, as elephants have dichromatic colour vision which prevents them from seeing shades of red and green (Yokoyama et al. 2005), they may also have used the contrast between the different colours (Judd 1943; Morgan et al. 1992; Regan et al. 2001) and not the colours themselves when making their choices. Ultimately, irrespective of the cue that they may have used, our results indicate that the elephants were able to utilise visual cues linked to the different colours to make their decisions.

Where, our results highlight that elephants have the ability to use visual cues when making small-scale foraging decisions, it is important to remember that elephants have one of the most highly developed olfactory abilities of any mammal (Shoshani et al. 2005; Niimura et al. 2014), and previous studies have found that elephants rely heavily on olfactory cues when making foraging decisions both within and between patches (Schmitt et al. 2018, 2020; McArthur et al. 2019; Bester et al. 2023). As such, it is unlikely that they fully rely on visual cues when making small-scale foraging decisions. Rather, it is more likely that African elephants use a combination of olfactory and visual cues. This then would allow them to forage more efficiently and maximise their nutritional gains. However, further research is needed to determine which of these two senses elephants rely on more when making these small-scale foraging decisions (i.e., < 10 m).

Despite the valuable insights that our findings provide, our study could be limited to some degree by the number of individual elephants that we tested. This, however, was unavoidable due to the limited number of semi-tame African elephants available. Nevertheless, as clear patterns emerged from our experiments, we are confident with our conclusions. However, future research incorporating a larger number of individuals would help expand our understanding of elephant visual ability.

Visually, as the elephants in our study were only able to distinguish between the different colours at 10 m or less, we suggest that the visual cues associated with colour (e.g., brightness, contrast) may only be reliable for elephants when moving between closely spaced patches and trees, and when selecting specific plant parts within individual trees. However, as we did not train half the elephants to select the canvases that indicated the lower quality food, our results are unable to determine whether the elephants were predisposed to selecting the visual cues associated with high-quality vegetation. Yet, as the elephants used in our experiments freely feed across the landscape and thus encounter these visual cues daily, it is possible that prior to our experiments that the elephants had already made an association between the small-scale visual cues that we tested and leaf quality. Nevertheless, the inability of the elephants to distinguish between the different colours at > 10 m suggests that the visual cues associated with leaf colour are unlikely to be driving the large-scale movements of elephants across the landscape.

## Electronic supplementary material

Below is the link to the electronic supplementary material.

Supplementary Material 1

## Data Availability

The data for this work were deposited into the Institutional Repository of the University of Pretoria. Moreover, they are freely available from the authors.

## References

[CR1] Aerts R (1996) Nutrient resorption from senescing leaves of perennials: are there general patterns? J Ecol 84:597–608. 10.2307/2261481

[CR2] Andersson S, Amundsen T (1997) Ultraviolet colour vision and ornamentation in bluethroats. Proc R Soc B Biol Sci 264:1587–1591. 10.1098/rspb.1997.0221

[CR3] Ballinger GA (2004) Using generalized estimating equations for longitudinal data analysis. Organ Res Methods 7:127–150. 10.1177/1094428104263672

[CR4] Bennett AT, Cuthill IC, Partridge JC, Maier EJ (1996) Ultraviolet vision and mate choice in zebra finches. Nature 380:433–435. 10.1038/380433a0

[CR6] Bester T, Schmitt MH, Shrader AM (2023) The deterrent effects of individual monoterpene odours on the dietary decisions of African elephants. Anim Cogn 26:1–15. 10.1007/s10071-023-01755-4 36800131 10.1007/s10071-023-01755-4 PMC10066090

[CR7] Bloom AJ, Chapin SF, Mooney HA (1985) Resource limitation in plants - An economic analogy. Annu Rev Ecol Syst 16:363–392. 10.1146/annurev.es.16.110185.002051

[CR8] Bowmaker JK (1980) Colour vision in birds and the role of oil droplets. Trends Neurosci 3:196–199. 10.1016/0166-2236(80)90072-7

[CR9] Bowmaker JK (1998) Evolution of colour vision in vertebrates. Eye 12:541–547. 10.1038/eye.1998.143 9775215 10.1038/eye.1998.143

[CR10] Caine NG, Surridge AK, Mundy NI (2003) Dichromatic and trichromatic Callithrix geoffroyi differ in relative foraging ability for red-green color-camouflaged and non-camouflaged food. Int J Primatol 24:1163–1175. 10.1023/B:IJOP.0000005985.18112.25

[CR11] Caves EM, Brandley NC, Johnsen S (2018) Visual acuity and the evolution of signals. Trends Ecol Evol 33:358–372. 10.1016/j.tree.2018.03.001 29609907 10.1016/j.tree.2018.03.001

[CR12] Changizi MA, Zhang Q, Shimojo S (2006) Bare skin, blood and the evolution of primate colour vision. Biol Lett 2:217–221. 10.1098/rsbl.2006.0440 17148366 10.1098/rsbl.2006.0440 PMC1618887

[CR13] Choong MF, Lucas PW, Ong JSY et al (1992) Leaf fracture toughness and sclerophylly: their correlations and ecological implications. New Phytol 121:597–610. 10.1111/j.1469-8137.1992.tb01131.x

[CR14] Corney D, Haynes J-D, Rees G, Lotto RB (2009) The brightness of colour. PLoS ONE 4:e5091. 10.1371/journal.pone.0005091 19333398 10.1371/journal.pone.0005091 PMC2659800

[CR71] Cox K, McGhie T, White A et al (2004) Skin colour and pigment chnages during ripening of'Hass' avocado fruit. Postharvet Biol Tec 31:287–294. 10.1016/j.postharvbio.2003.09.008

[CR16] de Moraes PZ, Diniz P, Spyrides MHC, Pessoa DMA (2021) The effect of pelage, background, and distance on predator detection and the evolution of primate color vision. Am J Primatol 83:1–14. 10.1002/ajp.23230 10.1002/ajp.23230 33475188

[CR15] de Moraes PZ, Diniz, Pessoa DMA (2023) Body posture, gaze, and predator detection: how stalking behaviour May influence colour vision evolution. BioRxiv 1–31. 10.1101/2023.05.30.542645

[CR17] Dominy NJ, Svenning JC, Li WH (2003) Historical contingency in the evolution of primate color vision. J Hum Evol 44:25–45. 10.1016/S0047-2484(02)00167-7 12604302 10.1016/s0047-2484(02)00167-7

[CR18] Duncan RD, Jenkins SH (1998) Use of visual cues in foraging by a diurnal herbivore, belding’s ground squirrel. Can J Zool 76:1766–1770. 10.1139/z98-119

[CR19] Eshelman BD, Jenkins SH (1989) Food selection by belding’s ground squirrels in relation to plant nutritional features. J Mammal 70:846–852. 10.2307/1381726

[CR20] Fairchild MD (2005) Color appearance models. John Wiley & Sons, Ltd. England

[CR21] Finger E, Burkhardt D (1994) Biological aspects of bird colouration and avian colour vision including ultraviolet range. Vis Res 34:1509–1514. 10.1016/0042-6989(94)90152-X 8023462 10.1016/0042-6989(94)90152-x

[CR70] Grunow J, Groenveld H, DuToit S (1980) Above-ground dry matter dynamics of the grass layer of a South African tree savanna. BES 68:877–889. 10.2307/2259463

[CR22] Hecht S (1949) Brightness, visual acuity, and colour blindness. Doc Opthalmologica 3:289–306. 10.1007/BF00162607 10.1007/BF00162607 18142208

[CR24] Hirata M, Kusatake N (2020) How cattle discriminate between green and dead forages accessible by head and neck movements by means of senses: reliance on vision varies with the distance to the forages. Anim Cogn 23:405–414. 10.1007/s10071-019-01344-4 31915949 10.1007/s10071-019-01344-4

[CR23] Hirata M, Arimoto C, Hattori N, Anzai H (2019) Can cattle visually discriminate between green and dead forages at a short distance while moving in the field? Anim Cogn 22:707–718. 10.1007/s10071-019-01268-z 31127432 10.1007/s10071-019-01268-z

[CR76] IBM Corp. Released 2020. IBM SPSS Statistics for Windows, Version 27.0. Armonk, NY: IBM Corp.

[CR72] Jachmann H, Bell R (1985) Utilization by elephants of the Brachystegia woodlands of the Kasungu National Park, Malawi. Afr J Ecol 23:245–258. 10.1111/j.1365-2028.1985.tb00955.x

[CR26] Jacobs GH (1983) Colour vision in animals. Endeavour 7:137–140. 10.1016/0160-9327(83)90006-6 6196176 10.1016/0160-9327(83)90006-6

[CR25] Jacobs GH (1993) The distribution and nature of colour vision among the mammals. Biol Rev Camb Philos Soc 68:413–471 8347768 10.1111/j.1469-185x.1993.tb00738.x

[CR27] Jacobs GH (2009) Evolution of colour vision in mammals. Philos Trans R Soc B Biol Sci 364:2957–2967. 10.1098/rstb.2009.0039 10.1098/rstb.2009.0039 PMC2781854 19720656

[CR28] Jacobs GH, Rowe MP (2004) Evolution of vertebrate colour vision. Clin Exp Optom 87:206–216. 10.1111/j.1444-0938.2004.tb05050.x 15312024 10.1111/j.1444-0938.2004.tb05050.x

[CR29] Johnsen S (2012) The optics of life: A biologists guide to light in nature. Princeton University Press, Princeton, NJ

[CR30] Judd DB (1943) Colour blindness and the detection of camouflage. Sci (80-) 97:544–546. 10.1126/science.97.2529.544 10.1126/science.97.2529.544 17773800

[CR31] Kelber A (2019) Bird colour vision– from cones to perception. Curr Opin Behav Sci 30:34–40. 10.1016/j.cobeha.2019.05.003

[CR32] Killingbeck KT (1996) Nutrients in senesced leaves: keys to the search for potential resorption and resportion proficiency. Ecology 77:1716–1727. 10.2307/2265777

[CR33] Lajtha K (1987) Nutrient reabsorption efficiency and the response to phosphorus fertilization in the desert shrub Larrea tridentata. Biogeochemistry 4:265–276. 10.1007/BF02187370

[CR34] Loarie SR, van Aarde RJ, Pimm SL (2009) Elephant seasonal vegetation preferences across dry and wet savannas. Biol Conserv 142:3099–3107. 10.1016/j.biocon.2009.08.021

[CR35] Lucas PW, Darvell BW, Lee PK et al (1995) The toughness of plant cell walls. Philos Trans R Soc B Biol Sci 348:363–372. 10.1098/rstb.1995.0074

[CR36] Lucas PW, Darvell BW, Lee PKD et al (1998) Colour cues for leaf food selection by Long-Tailed macaques (Macaca fascicularis) with a new suggestion for the evolution of trichromatic colour vision. Folia Primatol 69:139–152. 10.1159/000021576 10.1159/000021576 9595683

[CR38] Ma Y, Mazumdar M, Memtsoudis SG (2012) Beyond repetaed-measures analysis of variance: advanced statistical methods for the analysis of longitudinal data in anesthesia research. Reg Anesth Pain Med 37:99–105. 10.1097/AAP.0b013e31823ebc74 22189576 10.1097/AAP.0b013e31823ebc74 PMC3249227

[CR37] Macandza VA, Owen-Smith N, Cross PC (2004) Forage selection by African Buffalo in the late dry season in two landscapes. Afr J Wildl Res 34:113–121

[CR39] Maunders MJ, Brown SB (1983) The effect of light on chlorophyll loss in senescing leaves of sycamore (Acer Pseudoplatanus L). Planta 158:309–311. 10.1007/BF00397332 24264750 10.1007/BF00397332

[CR40] McArthur C, Finnerty PB, Schmitt MH et al (2019) Plant volatiles are a salient cue for foraging mammals: elephants target preferred plants despite background plant odour. Anim Behav 155:199–216. 10.1016/j.anbehav.2019.07.002

[CR41] McNaughton SJ (2013) Ecology of a grazing ecosystem: the Serengeti. Ecol Monogr 55:259–294. 10.2307/1942578

[CR79] Medlicott AP, Bhogal M, Reynolds SB (1986) Changes in peel pigmentation during ripening of mango fruit (Mangifera indica var. Tommy Atkins). Ann Appl Biol 109:651–656. 10.1111/j.1744-7348.1986.tb03222.x

[CR42] Melin AD, Chiou KL, Walco ER et al (2017a) Trichromacy increases fruit intake rates of wild capuchins (Cebus capucinus imitator). Proc Natl Acad Sci U S A 114:10402–10407. 10.1073/pnas.1705957114 28894009 10.1073/pnas.1705957114 PMC5625910

[CR43] Melin AD, Khetpal V, Matsushita Y et al (2017b) Howler monkey foraging ecology suggests convergent evolution of routine trichromacy as an adaptation for folivory. Ecol Evol 7:1421–1434. 10.1002/ece3.2716 28261454 10.1002/ece3.2716 PMC5330884

[CR44] Menzel R (1979) Spectral sensitivity and color vision in invertebrates. In: Autrum H (ed) Comparative physiology and evolution of vision in invertebrates. Handbook of sensory physiology. Springer, Berlin, Heidelberg, pp 503–580

[CR78] Mikulic-Petkovsek M, Rescic J, Schmitzer V, Stampar F, Slatnar A, Koron D, Veberic R (2015) Changes in fruit quality parameters of four Ribes species during ripening. Food Chem 173:363–374. 10.1016/j.foodchem.2014.10.011 10.1016/j.foodchem.2014.10.011 25466034

[CR45] Morgan MJ, Adam A, Mollon JD (1992) Dichromats detect colour-camouflaged objects that are not detected by trichromats. Proc R Soc B Biol Sci 248:291–295. 10.1098/rspb.1992.0074 10.1098/rspb.1992.0074 1354367

[CR46] Naseri P, Majd HA, Kariman N, Sourtiji A (2016) Comparison of generalized estimating equations (GEE), mixed effects models (MEM) and repeated measures ANOVA in analysis of menorrhagia data. J Paramed Sci 7:32–40. 10.22037/JPS.V7I1.11250

[CR81] Niimura Y, Matsui A, Touhara K (2014) Extreme expansion of the olfactory receptor gene repertoire in African elephants and evolutionary dynamics of orthologous gene groups in 13 placental mammals. Genome Res 24:1485–1496. 10.1101/gr.169532.113 10.1101/gr.169532.113 PMC4158756 25053675

[CR47] Owen-Smith N, Chafota J (2012) Selective feeding by a megaherbivore, the African elephant (Loxodonta africana). J Mammal 93:698–705. 10.1644/11-MAMM-A-350.1

[CR75] Pettigrew J, Bhagwandin A, Haagensen M, Manger PR (2010) Visual acuity and heterogeneities of retinal l ganglion cell densities and the tapetum lucidum of the African elephant (Loxodonta africana). Brain Behav Evol 75:251–261. 10.1159/000314898 10.1159/000314898 20587993

[CR73] Pretorius Y, Stigter J, de Boer W et al (2012) Diet selection of African elephant over time shows changing optimization currency. Oikos 121:2110–2120. 10.1111/j.1600-0706.2012.19680.x

[CR48] Regan BC, Julliot C, Simmen B et al (2001) Fruits, foliage and the evolution of primate colour vision. Philos Trans R Soc B Biol Sci 356:229–283. 10.1098/rstb.2000.0773 10.1098/rstb.2000.0773 PMC1088428 11316480

[CR50] Rensch B (1956) Increase of learning capability with increase of brain size. Am Nat 90:81–95. 10.1086/281911

[CR49] Rensch B (1957) The intelligence of elephants. Sci Am 196:44–49. https://doi.org/www.jstor.org/stable/24941883

[CR51] Saito A, Mikami A, Kawamura S (2005) Advantage of dichromats over trichromats in discrimination of colour-camouflaged stimuli on nonhuman primates. Am J Primitology 67:425–436. 10.1002/ajp.20197 10.1002/ajp.20197 16342068

[CR52] Savage A, Rice JM, Brangan JM et al (1994) Performance of African elephants (Loxodonta africana) and California sea lions (Zalophus californianus) on a two-choice object discrimination task. Zoo Biol 13:69–75. 10.1002/zoo.1430130109

[CR55] Schmitt MH, Ward D, Shrader AM (2016) Incorporating secondary metabolites, tannin-binding proteins, and diet breadth into carrying-capacity models for African elephants. Ecol Modell 332:8–18. 10.1016/j.ecolmodel.2016.03.016

[CR54] Schmitt MH, Shuttleworth A, Ward D, Shrader AM (2018) African elephants use plant odours to make foraging decisions across multiple Spatial scales. Anim Behav 141:17–27. 10.1016/j.anbehav.2018.04.016

[CR53] Schmitt MH, Shuttleworth A, Shrader AM, Ward D (2020) The role of volatile plant secondary metabolites as pre-ingestive cues and potential toxins dictating diet selection by African elephants. Oikos 129:24–34. 10.1111/oik.06665

[CR56] Seloana MQ, Jordaan JJ, Potgieter MJ, Kruger JW (2017) Feeding patterns of elephants at the Atherstone collaborative nature reserve. Afr J Ecol 56:445–454. 10.1111/aje.12422

[CR77] Shrader AM, Owen-Smith N, Ogutu JO (2006). How a mega-grazer copes with the dry season: food and nutrient intake rates by white rhinoceroses in the wild. Funct Ecol 20:376–384. 10.1111/j.1365-2435.2006.01107.x

[CR57] Shlaer S (1937) The relation between visual acuity and illumination. J Gen Physiol 21:165–188. 10.1085/jpg.21.2.165 19873045 10.1085/jgp.21.2.165 PMC2141937

[CR58] Shrader AM, Bell C, Bertolli L, Ward D (2012) Forest or the trees: at what scale do elephants make foraging decisions? Acta Oecol 42:3–10. 10.1016/j.actao.2011.09.009

[CR59] Shyan-Norwalt MR, Peterson J, King BM et al (2010) Initial findings on visual acuity thresholds in an African elephant (Loxodonta africana). Zoo Biol 29:30–35. 10.1002/zoo.20259 19598240 10.1002/zoo.20259

[CR60] Smith AC, Buchanan-smith HM, Surridge AK et al (2003) The effect of colour vision status on the detection and selection of fruits by tamarins (Saguinus spp). J Exp Biol 206:3159–3165. 10.1242/jeb.00536 12909697 10.1242/jeb.00536

[CR61] Stavenga DG (2002) Colour in the eyes of insects. J comp physiol A neuroethol sensory, neural. Behav Physiol 188:337–348. 10.1007/s00359-002-0307-9 10.1007/s00359-002-0307-9 12073079

[CR62] Stone J, Halasz P (1989) Topography of the retina in the elephant Loxodonta africana. Brain Behav Evol 34:84–95. 10.1159/000116494 2819413 10.1159/000116494

[CR63] Suedmeyer K (2006) Special senses. In: Fowler ME, Mikota SK (eds) Biology, medicine and surgery of elephants. Blackwell Publishing, Ames, IA, pp 399–407

[CR64] Sumner P, Mollon JD (2000) Catarrhine photopigments are optimized for detecting targets against a foliage background. J Exp Biol 203:1963–1986. 10.1242/jeb.203.13.1963 10851115 10.1242/jeb.203.13.1963

[CR65] Thoma DP, Bailey DW, Long DS et al (2002) Short-term monitoring of rangeland forage conditions with AVHRR imagery. J Range Manag 55:383–389. 10.2458/azu_jrm_v55i4_thoma

[CR66] Thüs P, Lunau K, Wester P (2020) Colour vision in Sengis (Macroscelidea, afrotheria, Mammalia): Choice experiments indicate dichromatism. Behaviour 157:1127–1151. 10.1163/1568539X-bja10039

[CR67] Warrant E, Somanathan H (2022) Colour vision in nocturnal insects. Philos Trans R Soc B Biol Sci 377:20210285. 10.1098/rstb.2021.0285 10.1098/rstb.2021.0285 PMC9441242 36058247

[CR68] Wood M, Chamaillé S, Hammerbacher A, Shrader AM (2022) African elephants can detect water from natural and artificial sources via olfactory cues. Anim Cogn 25:53–61. 10.1007/s10071-021-01531-2 34292432 10.1007/s10071-021-01531-2

[CR74] Yang S, Walther G, Weng (2015) Stop and smell the pollen: The role olfaction and vision of the oriental honey buzzard in identifying food. PLos ONE 10:1–18. 10.1371/journal.pone.0130191 10.1371/journal.pone.0130191 PMC4503435 26177533

[CR69] Yokoyama S, Takenaka N, Agnew DW, Shoshani J (2005) Elephants and human color-blind deuteranopes have identical sets of visual pigments. Genetics 170:335–344. 10.1534/genetics.104.039511 15781694 10.1534/genetics.104.039511 PMC1449733

