# Supplementary material for: Can African elephants use leaf colour as a visual cue when making foraging decisions?
Source: Anim Cogn. 2025 Jun 13;28(1):46. doi: 10.1007/s10071-025-01972-z (PMC12166006; doi:10.1007/s10071-025-01972-z)
Supplement: Supplementary file 1 — Supplementary Material 1 [file 10071_2025_1972_MOESM1_ESM.docx]

**Do African elephants use leaf colour as a visual cue when making foraging decisions?**

Claire L. Peinke* and Adrian M. Shrader

Department of Zoology & Entomology, University of Pretoria. Private Bag X28, Pretoria 0028, South Africa

*Corresponding author: clairepeinke@gmail.com

ORCID IDs:

Claire L. Peinke: 0000-0003-4525-3653

Adrian M. Shrader: 0000-0002-6451-6132

**Online Resource 1**

**Training**

The elephants were able to distinguish between the colour canvases over the entire four-week (30 days) training period (GEE: χ^2^ = 127202912.62, df = 2, p < 0.0001; Figure S1).


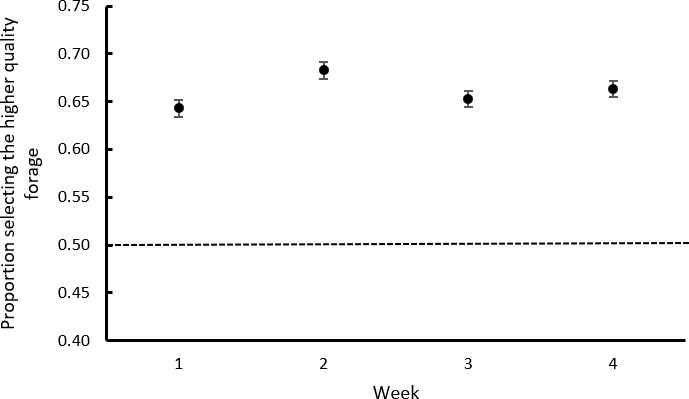


**Fig. OR1** Mean proportion of choices where the elephants selected the higher quality forage colour over the four-week (30 days) training period. Marginal means (+95% CI) of the proportion of the selection for each week are plotted. Overlap of the error bars with the expected 0.5 (dashed line) shows no preference (i.e., random selection). Error bars above the 0.5 expected shows preference for the higher-quality forage, while error bars below the dashed line shows preference for the lower quality forage
